# Pretreatment Fasting Glucose and Insulin as Determinants of Weight Loss on Diets Varying in Macronutrients and Dietary Fibers—The POUNDS LOST Study

**DOI:** 10.3390/nu11030586

**Published:** 2019-03-11

**Authors:** Mads F. Hjorth, George A. Bray, Yishai Zohar, Lorien Urban, Derek C. Miketinas, Donald A. Williamson, Donna H. Ryan, Jennifer Rood, Catherine M. Champagne, Frank M. Sacks, Arne Astrup

**Affiliations:** 1 Department of Nutrition, Exercise and Sports, Faculty of Sciences, University of Copenhagen, Rolighedsvej 26, 1958 Frederiksberg, Denmark; ast@nexs.ku.dk; 2 Pennington Biomedical Research Center of the Louisiana State University System, Baton Rouge, LA 70803, USA; George.Bray@pbrc.edu (G.A.B.); Derek.Miketinas@pbrc.edu (D.C.M.); WilliaDA@pbrc.edu (D.A.W.); ryandh@pbrc.edu (D.H.R.); Jennifer.Rood@pbrc.edu (J.R.); Catherine.Champagne@pbrc.edu (C.M.C.); 3 Gelesis, Boston, MA 02116, USA; yzohar@gelesis.com (Y.Z.); lurban@gelesis.com (L.U.); 4 School of Nutrition and Food Sciences, Texas Woman’s University, Denton, TX 76204, USA; 5 Nutrition Department, Harvard T.H Chan School of Public Health and Department of Medicine, Harvard Medical School, Boston, MA 02115, USA; fsacks@hsph.harvard.edu

**Keywords:** glucose, insulin, weight, diet, macronutrient composition, clinical nutrition

## Abstract

Efforts to identify a preferable diet for weight management based on macronutrient composition have largely failed, but recent evidence suggests that satiety effects of carbohydrates may depend on the individual’s insulin-mediated cellular glucose uptake. Therefore, using data from the POUNDS LOST trial, pre-treatment fasting plasma glucose (FPG), fasting insulin (FI), and homeostatic model assessment of insulin resistance (HOMA-IR) were studied as prognostic markers of long-term weight loss in four diets differing in carbohydrate, fat, and protein content, while assessing the role of dietary fiber intake. Subjects with FPG <100 mg/dL lost 2.6 (95% CI 0.9;4.4, *p* = 0.003) kg more on the low-fat/high-protein (*n* = 132) compared to the low-fat/average-protein diet (*n* = 136). Subjects with HOMA-IR ≥4 lost 3.6 (95% CI 0.2;7.1, *p* = 0.038) kg more body weight on the high-fat/high-protein (*n* = 35) compared to high-fat/average-protein diet (*n* = 33). Regardless of the randomized diet, subjects with prediabetes and FI below the median lost 5.6 kg (95% CI 0.6;10.6, *p* = 0.030) more when consuming ≥35 g (*n* = 15) compared to <35 g dietary fiber/10 MJ (*n* = 16). Overall, subjects with normal glycemia lost most on the low-fat/high-protein diet, subjects with high HOMA-IR lost most on the high-fat/high protein diet, and subjects with prediabetes and low FI had particular benefit from dietary fiber in the diet.

## 1. Introduction

During the past 30 years, there has been a great deal of controversy about the composition of the optimal diet for weight loss and maintenance. Some have defended the more conventional low-fat/high-carbohydrate diet [1,2], whereas others point to a restriction in carbohydrates as being more effective [3]. Numerous strategies for modifying carbohydrate intake have been proposed, from ketogenic very-low-carbohydrate diets [4] to diets with increased protein and a lowered glycemic index (GI) of the carbohydrates [5]. Efforts to identify a preferable diet for weight loss and weight loss maintenance based on macronutrient composition have largely failed [6], implying that no single diet is ideal for all participants with overweight and obesity [7]. On the other hand, dietary fiber is regarded as important for weight regulation [8], as diets including more fruits, vegetables and whole grains are associated with lower body weight in randomized dietary studies [9,10], as well as in observational studies [8].

The glucostatic hypothesis suggests that the central nervous system monitors blood glucose as part of the established appetite regulatory system. As eating progresses, glucose in the blood increases, leading to increased hypothalamic glucose utilization, ultimately causing the individual to become satiated and to stop eating [11]. Recently, the glucose uptake in the brain during a hyperglycemic clamp, simulating postprandial levels, was found to be reduced in individuals with obesity compared to normal weight subjects and even more so in patients with type 2 diabetes [12]. Furthermore, brain glucose uptake was positively correlated with fullness and satiety [12]. Collectively, this suggests that whether or not a carbohydrate-rich diet, which, in the case of high glycemic index foods, would result in rapid increases in blood glucose, should be recommended as diets for weight loss and weight loss maintenance depends on the degree to which glucose enters the brain. Carbohydrate-rich meals may, therefore, be satiating in insulin-sensitive individuals, but less so in more insulin-resistant individuals. Individuals with prediabetes or type 2 diabetes may instead depend more on dietary fiber intake to stimulate satiety and improve glycemic control [13], as well as other satiety hormones, such as CCK, GLP-1, and PYY, which are released mainly in response to fats and protein reaching the small intestine [8].

Based on stratified analysis of several studies summarized in a recent review [14], it was proposed that diets low in fat and high in protein will work best for individuals with normal fasting glucose and diets high in dietary fibers will work particularly well among subjects with impaired fasting glucose. The POUNDS LOST study offers the opportunity to test this hypothesis, since it randomized subjects to four diets varying in macronutrients with a minimum of 20 g of dietary fiber per day. In the main paper, there were no differences in weight loss between the randomized groups [15]. However, the potential role of pretreatment fasting plasma glucose (FPG) and fasting insulin (FI) on weight loss according to randomized diets and dietary fiber intake was not investigated, and is the subject of this paper.

The purpose of this study was to analyze data from the POUNDS LOST trial [15], to investigate whether FPG and FI are prognostic markers for long-term weight loss in four diets differing in carbohydrate, fat, and protein content, and assess the role of dietary fiber intake. We hypothesized that normoglycemic subjects would lose more weight on the low-fat/high-protein diet, that dietary fiber intake would be positively associated with weight loss particularly among subjects with prediabetes, and that those being most insulin resistance would lose more weight on the high-fat/high-protein diet.

## 2. Materials and Methods

In the original trial, 811 overweight adults were randomized to one of four energy-reduced diets (deficit of 750 kcal per day from baseline) varying in macronutrient composition for 24 months with the goals for all groups of having at least 20 g of dietary fiber per day while recommending carbohydrates with a low glycemic index. The nutrient goals for the four diet groups were: 20% fat, 15% protein, and 65% carbohydrates (low-fat/average-protein); 20% fat, 25% protein, and 55% carbohydrates (low-fat/high-protein); 40% fat, 15% protein, and 45% carbohydrates (high-fat/average-protein); and 40% fat, 25% protein, and 35% carbohydrates (high-fat/high-protein). At baseline, FPG and FI were measured from which homeostatic model assessment of insulin resistance (HOMA-IR) was calculated, and participants were asked to complete a 5-day diet record. Body weight was measured at baseline and after 24 months of intervention. After 6 and 24 months, 24-h dietary recalls were collected during telephone interviews on 3 nonconsecutive days in a random sample of 50% of the participants. The proportion of attended counseling sessions for weight loss during the 24 months was calculated as sessions attended divided by sessions offered and split into high and low attendance using the median value (0.44). Among others, the presence of diabetes was a criterion for exclusion. Detailed information about the study has been published [15]. The study was approved by the human subjects committee and by the data and safety monitoring board. All participants gave written informed consent. The study was registered on clinicaltrials.gov with the identifier: NCT00072995.

For this re-analysis, baseline FPG levels were used to stratify subjects as being normoglycemic (FPG < 100 mg/dL) or prediabetic (FPG ≥ 100 mg/dL, NOTE: no upper limit as diabetes was a criterion for exclusion) through the use of the FPG cutoffs published by the American Diabetes Association [16], as pre-treatment FPG was recently shown to determine weight loss and weight loss maintenance success to diets varying in macronutrient composition and fiber content [14]. Furthermore, median FI concentration among subjects with prediabetic having dietary records (13.8 µIU/mL) was used to dichotomize subjects into low and high FI in accordance with previous procedures in which cut-offs ranging between 10.5 and 13 µIU/mL were used [17]. Similarly, median HOMA-IR value among subjects with prediabetic having dietary records (4.0) was used to dichotomize subjects into low and high HOMA-IR. Subjects were included in the current study if they had a baseline measure of FPG and FI as well as a 24-month measurement of body weight. Dietary fiber intake during the 24 months was calculated as mean intake at 6 and 24 months expressed as g/10 MJ (~2400 kcal). If the 24-h recall was missing at either 6 or 24 months, the other constituted the mean value. Furthermore, changes in dietary fiber intake (g/10 MJ) were calculated using the mean intake during the intervention by subtracting the baseline fiber intake.

Descriptive characteristics of the study population (completers only) are presented as mean ± SD or as proportions (%) and differences between glycemic groups was tested using one-way ANOVA or Pearson’s chi-squared test. Differences in weight change between FPG and FI groups (and the combination of the two using an interaction term) were analyzed by means of linear mixed models. The linear mixed models comprised fixed effects including age, gender, and baseline BMI and site as random effect. Results are shown as 24-month mean weight change from baseline with 95% confidence interval (CI). Differences in weight change from baseline between diets were compared within each blood marker group through the use of pairwise comparisons with post hoc *t*-tests. The 24-month weight change according to self-reported dietary fiber intake (during the intervention and as changes from baseline to intervention) in the overall population, as well as in selected groups based on FPG and FI groups (and the combination of the two) were reported as Pearson correlation coefficients and partial correlation coefficients adjusting for age, gender, and baseline BMI. Finally, the difference in 24-month weight loss between participants consuming ≥35 g fiber/10 MJ and <35 g fiber/10 MJ during the intervention was compared using *t*-tests. The level of significance was set at P < 0.05, with no adjustment for multiple testing, and statistical analyses were conducted using STATA/SE 14.1 (Houston, TX, USA).

## 3. Results

The 639 subjects used for these analyses included participants who were 61% women, were 52 ± 9 years of age and had a BMI of 32.7 ± 3.8 kg/m^2^. Differences in age, BMI, weight and gender distribution (*p* ≤ 0.008), but not in completion rate (*p* = 0.81), were observed between the FPG/FI subgroups (Table 1). In addition, among the 317 subjects having valid dietary records, total energy intake, dietary fat and fiber intake (*p* ≤ 0.035), but not carbohydrate and protein intake (*p* ≥ 0.22), varied slightly between FPG/FI subgroups at baseline (Table 1).

Overall, the low-fat/high-protein diet (*n* = 157) produced a 1.8 (95% CI 0.2;3.4, *p* = 0.03) kg greater weight loss compared to the low-fat/average-protein diet (*n* = 166) (Table 2). This difference was 2.6 (95% CI 0.9;4.4, *p* = 0.003) kg among subjects with normoglycemic and −1.4 (95% CI −5.3;2.4, *p* = 0.46) kg among subjects with prediabetes [mean difference: 4.1 kg (95% CI −0.1;8.3, *p* = 0.057)]. This indicates that glycemic status modulates the effect of a low-fat, high-protein diet on weight loss over 2 years. The diet appears more effective in those with normoglycemia than prediabetes. Further subdividing the normoglycemia group showed that this difference was 2.9 (95% CI 0.9;4.9, *p* = 0.005) kg among subjects with normoglycemia and low FI, and 2.1 (95% CI −1.4;5.6, *p* = 0.25) kg among subjects with normoglycemia and high FI [mean difference: 0.8 kg (95% CI −3.3;4.8, *p* = 0.71).

Furthermore, subjects with high HOMA-IR lost 3.6 (95% CI 0.2;7.1, *p* = 0.038) kg more body weight on the high-fat/high-protein diet compared to high-fat/average-protein diet, whereas this difference was −0.9 (95% CI −2.7;0.9, *p* = 0.32) kg among subjects with low HOMA-IR [mean difference: 4.5 (95% CI 0.7;8.4, *p* = 0.022)].

Independent of the type of diet, subjects attending at least 44 percent (median value) of the counseling sessions lost 5.6 (95% CI 4.5;6.6, *p* < 0.001) kg more compared to those attending fewer sessions (−6.9 vs. −1.3 kg). Among this subgroup with attendance above the median, subjects who had high HOMA-IR lost 4.8 kg (95% CI 0.01;9.6, *p* = 0.049) more body weight when randomized to the high-fat/high-protein diet compared to the high-fat/average-protein, whereas this difference was −1.1 (95% CI −3.5;1.2, *p* = 0.35) kg among subjects with low HOMA-IR [mean difference: 5.9 (95% CI 0.6;11.3, *p* = 0.029)] (Table S1).

The self-reported dietary fiber intake was 9.8 (95% CI 8.7;11.0, SD 10.4, *n* = 317, *p* < 0.001) g/10 MJ higher during the intervention compared to the baseline. The fiber intake mainly came from the carbohydrate-rich foods, so fiber intake was highest in the low-fat/average-protein diet and lowest in the high-fat/high-protein diet and intermediate in the two remaining diets (Table 3).

Overall, differences in dietary fiber intake between baseline and intervention, as well as fiber intake during the intervention, were negatively correlated with weight change (r = −0.17 to −0.23, *p* ≤ 0.002). This negative correlation existed for most subgroups of FPG, FI, and HOMA-IR but was most pronounced among subjects with prediabetes and low FI (r = −0.45 to −0.47, *p* ≤ 0.011) (Table 4). This correlation remained significant (r = −0.40, *p* = 0.047) after additionally adjusting for fat, protein and carbohydrate intake during the intervention.

**Figure 1:**
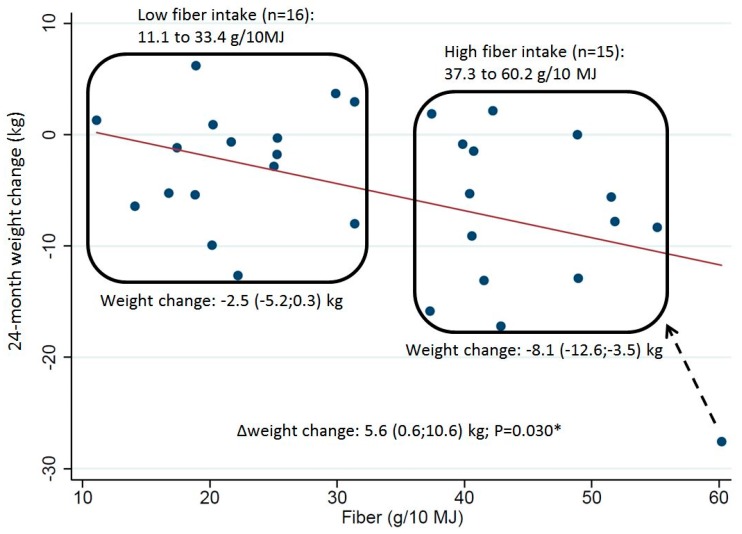
Dietary fiber intake as a function of weight change among subjects with prediabetes and low fasting insulin. Values for fiber intake is reported as range and weight change is reported as mean (95%CI). *Analyzed using *t*-test. When excluding the one subject with self-reported fiber intake of 60 g/10 MJ the *p*-value was *p* = 0.057.

Subjects consuming ≥35 g dietary fiber/10 MJ during the intervention lost 2.4 kg (95% CI 0.6;4.1, *p* = 0.008) more compared to those consuming <35 g dietary fiber/10 MJ. This difference existed for most subgroups of FPG, FI, and HOMA_IR, but was most pronounced among subjects with prediabetes and low FI [5.6 kg (95% CI 0.6;10.6, *p* = 0.030)] (Table 4 and Figure 1).

## 4. Discussion

As hypothesized, we found that subjects with normoglycemia lost the most body weight when randomized to the low-fat/high-protein diet, and that subjects with high HOMA-IR lost the most on the high-fat/high-protein diet. Furthermore, we found that participants with the highest intake of dietary fiber lost more body weight during the 24-month dietary intervention period, which was particularly evident among individuals with prediabetes and having below median of FI, where a 5.6 kg difference was observed among those below and above the median of fiber intake. These results are in accordance with previous studies that found that participants with normal FPG lose approximately 1 kg more during a 3–6 month period on a low fat diet [14,17,18]. Participants with impaired fasting glucose lose approximately 4 kg more weight than participants with normal glycaemia over 6 months when exposed to diets higher in dietary fibers [14,17,19]. Furthermore, participants with pretreatment FPG ≥ 126 mg/dL have been found to lose approximately 2 kg more body weight after 5 years on a Mediterranean diet compared to subjects with normoglycemia [14,20].

Satiety is a multi-factor construct that is more than just the metabolic effects of nutrients in the gut and intestine. It also includes cognitive and sensory signals generated by the sight, smell, and taste of foods [21]. However, satiety research has typically looked at the physiological effects of food ingredients, where protein exerts satiety through gastrointestinal hormonal signaling by e.g., GLP-1 and PYY, and dietary fiber (dependent of fiber type) provides satiety through increased viscosity, gelling in the stomach, replacing of energy dense foods, and fermentation in the gut are thought to positively affect satiety [13]. Recent evidence confirms the importance of fermentation of dietary fiber by the microbiota to facilitate energy homeostasis possibly through succinate and short chain fatty acids activating intestinal gluconeogenesis signaling to the brain by gastrointestinal nerves [22]. In addition, furthermore, different sources of dietary fiber should be matched with existing microbiota composition of the individual in order to lower body weight [23,24] and improve glucose metabolism [25], as proposed in a recent review stratifying subjects in enterotypes [26]. Furthermore, for non-diabetics, carbohydrates are considered more satiating compared to fats, suggesting that foods should be high in proteins, carbohydrates, and dietary fibers to have the optimal effect on appetite control [21]. However, emerging evidence suggests that carbohydrates will affect satiety primarily among subjects with normoglycemia and, to a lesser extent, among subjects with prediabetes, as less blood glucose will enter the brain (and perhaps other relevant tissues) to generate satiety signals [12]. This concept is supported by the present study, finding that a low-fat/high-protein diet is marginally better among subjects with normoglycemia as long as the glycemic load is keep down and fiber content kept at a minimum (recommending low GI carbohydrates and minimum 20 g/day of dietary fibers), and that a high-fat/high-protein diet is marginally better among the most insulin resistant subjects. These findings support the hypothesis that people with prediabetes/insulin resistance should to a larger extent rely on satiety hormones that are released mainly in response to fats and protein [for example, cholecystokinin (CCK), glucagon-like peptide 1 (GLP-1), and peptide YY (PYY)] reaching the small intestine dietary fat [27] and/or dietary fiber, for glycaemia control [28] and to enable weight loss and combat weight regain [13]. Generally, higher protein intake, as assessed by urea nitrogen/creatinine, has previously been shown to produce larger weight loss after both 6 and 24 months in the present study, and supports our findings that protein contents seem to be important for all phenotypes [29].

Dietary fiber was not a part of the intervention study, and we therefore used self-reported dietary data to investigate the importance of dietary fiber in the subgroups of FPG and FI. Nevertheless, looking at the self-reported fiber intake, it seems as if the randomized diets could have been confounded by dietary fiber intake, as the diets with the highest amount of carbohydrate also had higher fiber intake (among the subgroup that reported their dietary intake). This was perhaps not the case for subjects with normoglycemia as the low fat/high protein diet produced higher weight loss compared to the low-fat/average-protein diet that contained more dietary fiber. However, among subjects with prediabetes, it seems as if dietary fiber is more important than the exact macronutrient composition of the diet at least when recommending low GI carbohydrates, as in the present study. This was seen especially among subjects with prediabetes and FI below the median, which is in good agreement with the three studies in reference [17], but not with all studies [19]. Once the prediabetic state is more advanced, as is likely the case for subjects with prediabetes and high FI, it seems that an adverse effect of carbohydrates overrules the potential beneficial effect of dietary fiber intake, indicating a need to replace carbohydrates in the diet with proteins and fats. This beneficial effect of a diet higher in fats and lower in carbohydrates was observed among subjects with high HOMA-IR in the present study and was observed among subjects with fasting glucose ≥126 mg/dL and among type 2 diabetes patient [14,17,20,30,31].

Compliance or adherence is a general problem in long-term dietary intervention studies, as is evident from the overall weight loss and weight regain usually seen after 6 months [32]. The substantial diminished adherence after the first months can also be seen in the present study as weight regain occurred after 6 months, even when being prescribed an energy-restricted diet [15]. In the present study, 750 kcal/day deficit diets were designed based on resting energy expenditure and activity level at baseline. Using the prediction equation developed by Kevin Hall and colleagues [33], subjects in the present study should have lost approximately 33 kg during this 2 year period, had they been adherent to the energy restriction. The actual weight loss was only 4 kg among the 80% completing the study. Overall, these findings suggest that participants in weight-loss programs revert to their customary energy intake, and most likely also macronutrient composition, over time. This lack of adherence makes it difficult to investigate predictors of weight loss from pretreatment personal characteristics. Therefore, a sensitivity analysis was carried out among the half attending the most counseling sessions as those were expected to adhere better to the diets. Although finding those subjects to have lost substantially more weight compared to those attending fewer sessions (as reported previously [15]) FPG, FI, and HOMA-IR were not better predictors of weight loss success on the different diets in this subgroup. Challenges with adherence to the diet were also noted in the 24 month CHO-study that nevertheless found some large differences among a relatively small group of prediabetic obese subjects [34]. Another example highlighting the importance of adherence is from a study that compared an ad libitum low-fat/medium-protein diet with a low-fat/high-protein diet for 24 months among a group of primarily normoglycemic subjects (89% had FPG <100 mg/dL) [35]. In fact, this is the same macronutrient composition found to produce marginally higher weight loss in the present study—especially among normoglycemic subjects. The first 6 months included a strictly controlled dietary intervention with full provision of food from a purpose-built shop where the low-fat/high-protein diet was found to produce a 3.5 kg (*p* = 0.008) greater weight loss. During the following 6 months, with dietary counseling only, this difference diminished to become an insignificant 1.9 kg. Finally, after an additional 12 months of follow-up, there was no difference between the diets. Therefore, food provision (or other innovative initiatives) that could increase adherence is probably the only way to examine the true effect of different diets on health [5,10,17,19,35]. However, this is very expensive, and cannot be sustained over prolonged periods of time. Therefore, if investigated over a prolonged period of time, it probably needs to be a familiar and common diet in the region. An example of this could be the Mediterranean diet that in Spain was recently found to predict the best weight loss outcome over 5 years among subjects with elevated fasting glucose at baseline [14,20]. In agreement with this, the high-fat diets in the present study have previously been associated with higher levels of dietary adherence compared to low-fat diets [36], likely because the high-fat diets resembled the baseline/habitual diets the most.

Despite the relatively low adherence to the energy-restricted diets, the infrequent dietary reporting, and the expected day-to-day variability in fasting glucose and insulin, we found some evidence for the use of FPG and FI as determinants of weight loss on different diets. However, evidence is still conflicted, and more research is needed. The evidence is incomplete, but it currently suggests that people without diabetes but with normal fasting glucose and insulin sensitivity can achieve marginally greater success on low-fat regimens, preferably higher in protein, provided very high GLs are avoided [14,17,18,35,37]. The results further suggest that people with impaired fasting glucose (especially when FI is low) should have their main dietary focus be the incorporation of more dietary fiber [14,17,19]. Finally, in those with insulin resistance, FPG ≥ 126 mg/dL, and type 2 diabetes patients could benefit from increasing protein and fat intake at the expense of carbohydrates [14,17,20,30,31]. These different dietary patterns still need to be compared in randomized trials where the adherence in real-life settings (behavioral support and cultural and social factors) should be removed to investigate the effect of the individual underlying biology. The concepts of personalized nutrition or personalized lifestyle may drive some innovative new research in the area of weight management.

The strengths of our study include the 2-year duration, the consistent findings for both changes and level of dietary fiber consumption during the intervention, and the post-hoc analysis of the study that ensured a completely unbiased observation, whereby neither the investigators nor the participants knew about the background or aim of the current re-analysis. On the other hand, the post-hoc testing involved a relatively large number of statistical comparisons within subgroups of the population, which increases the risk of false positives, as well as leading to an increased risk of failing to detect differences due to power.

## 5. Conclusions

This study identified modest differences in diet-specific weight loss between glycemic phenotypes, indicating that subjects with normoglycemia could benefit the most from low-fat/high-protein diets, subjects with prediabetes (and low insulin) could benefit the most from diets high in dietary fiber, and subjects with insulin resistance (high HOMA-IR) could benefit the most from high-fat/high-protein diets. However, these findings need to be confirmed in randomized trials with this aim as a primary end-point.

## Figures and Tables

**Table 1:** Baseline characteristics of the completing study populations stratified by fasting glucose and insulin.

	FPG < 100 mg/dL & FI < 13.8 µIU/mL	FPG < 100 mg/dL & FI ≥ 13.8 µIU/mL	FPG ≥100 mg/dL & FI < 13.8 µIU/mL	FPG ≥ 100 mg/dL & FI ≥ 13.8 µIU/mL	*p*-Value
All (*n* = 639)	386	136	50	67	
Completion (%)	78.6	79.5	82.0	82.7	0.81
Age	50.9 ± 9.4 ^a^	52.3 ± 8.4 ^ab^	55.0 ± 6.9 ^b^	53.1 ± 9.7 ^ab^	0.008
Sex, % females	68.9	53.7	46.0	43.3	<0.001
Body weight, kg	88.8 ± 14.6 ^a^	100.0 ± 14.7 ^bc^	95.3 ± 13.3 ^c^	101.4 ± 14.4 ^b^	<0.001
BMI, kg/m^2^	31.6 ± 3.7 ^a^	34.5 ± 3.6 ^c^	32.8 ± 3.8 ^b^	34.7 ± 3.1 ^c^	<0.001
Fasting glucose, mg/dL	88 (83;92)	92 (87;96)	106 (102;115)	108 (103;115)	
Fasting Insulin, µIU/mL	7.9 (6.1;10.5)	17.3 (15.4;21.8)	10.3 (8.3;11.9)	19.8 (16.7;26.2)	
HOMA-IR	1.7 (1.3;2.3) ^a^	3.9 (3.5;4.7) ^b^	2.8 (2.2;3.3) ^c^	5.4 (4.6;7.1) ^d^	<0.001
Diet record subgroup (*n* = 317)	179	75	31	32	
Age	52.3 ± 9.2	53.4 ± 8.6	55.1 ± 7.1	52.1 ± 8.8	0.38
Sex, % females	62.6	49.3	48.4	28.1	0.002
Body weight, kg	89.3 ± 15.8 ^a^	100.9 ± 14.3 ^b^	95.3 ± 14.2 ^b^	101.5 ± 14.4 ^b^	<0.001
BMI, kg/m^2^	31.4 ± 3.8 ^a^	34.3 ± 3.4 ^b^	32.6 ± 3.9 ^a^	34.7 ± 2.7 ^b^	<0.001
Fasting glucose, mg/dL	88 (83;92)	93 (87;96)	106 (103;110)	110 (106;119)	
Fasting Insulin, µIU/mL	8.1 (6.1;10.5)	17.2 (15.7;19.8)	10.3 (8.3;11.9)	19.1 (16.5;23.3)	
HOMA-IR	1.7 (1.3;2.3) ^a^	3.9 (3.5;4.5) ^b^	2.8 (2.2;3.3) ^c^	5.3 (4.6;6.9) ^d^	<0.001
Energy intake (kcal/day)	1976 ± 494 ^a^	2126 ± 579 ^b^	1856 ± 630 ^a^	2301 ± 647 ^b^	0.002
Carbohydrate (E%)	45.1 ± 7.7	44.7 ± 7.0	45.2 ± 8.4	41.9 ± 6.6	0.17
Fat (E%)	36.9 ± 6.0 ^ab^	37.9 ± 5.4 ^b^	35.4 ± 6.9 ^a^	39.3 ± 5.6 ^bc^	0.035
Protein (E%)	17.9 ± 3.4	17.4 ± 3.2	18.8 ± 3.8	17.6 ± 2.5	0.22
Fiber intake (g/day)	18.1 ± 7.3	16.9 ± 5.4	17.6 ± 5.4	17.9 ± 5.8	0.61
Fiber intake (g/10 MJ)	22.2 ± 7.7 ^a^	19.5 ± 5.7 ^b^	24.5 ± 10.1 ^a^	19.1 ± 6.3 ^b^	0.002

Abbreviations: BMI, Body mass index; E%, Energy percentage; FI, Fasting insulin; FPG, Fasting plasma glucose; HOMA-IR, Homeostatic model assessment of insulin resistance. Data are mean ± SD, median (IQR), and proportions. Tested by one-way ANOVA with different superscript letters within a row indicate significant differences (*p* < 0.05) or tested for overall difference by chi-square.

**Table 2:** Two-year weight change according to randomization and stratified on pretreatment FPG and FI (*n* = 639).

	LF-AP 65% Carb	LF-HP 55% Carb	∆ (LF-AP vs. LF-HP) Weight Change (kg)	HF-AP 45% Carb	HF-HP 34% Carb	∆ (HF-AP vs. HF-HP) Weight Change (kg)
All ^1^	(*n* = 166) −3.3 (−4.4; −2.1) ^a^	(*n* = 157) −5.0 (−6.2; −3.9) ^b^	1.8 (0.2;3.4) *	(*n* = 148) −4.0 (−5.2; −2.8) ^ab^	(*n* = 168) −4.0 (−5.1; −2.9) ^ab^	−0.03 (−1.7;1.6)
FPG < 100 mg/dL	(*n* = 136) −2.9 (−4.2; −1.7) ^a^	(*n* = 132) −5.6 (−6.8; −4.3) ^b^	2.6 (0.9;4.4) *	(*n* = 119) −4.2 (−5.5; −2.9) ^ab^	(*n* = 135) −3.9 (−5.1; −2.6) ^ab^	−0.4 (−2.1;1.4)
FI < 13.8 µIU/mL	(*n* = 97) −2.6 (−4.1; −1.2) ^a^	(*n* = 105) −5.5 (−6.9; −4.1) ^b^	2.9 (0.9;4.9) *	(*n* = 80) −4.5 (−6.1; −2.9) ^ab^	(*n* = 104) −3.6 (−5.0; −2.2) ^ab^	−0.9 (−3.0;1.2)
FI ≥ 13.8 µIU/mL	(*n* = 39) −3.7 (−5.9; −1.4)	(*n* = 27) −5.7 (−8.5; −3.0)	2.1 (−1.4;5.6)	(*n* = 39) −3.6 (−5.9; −1.3)	(*n* = 31) −4.7 (−7.3; −2.2)	1.1 (−2.3;4.5)
FPG ≥ 100 mg/dL	(*n* = 30) −4.6 (−7.2; −2.0)	(*n* = 25) −3.2 (−6.0; −0.3)	−1.4 (−5.3;2.4)	(*n* = 29) −2.4 (−5.1;0.2)	(*n* = 33) −4.4 (−6.9; −2.0)	2.0 (−1.6;5.6)
FI < 13.8 µIU/mL	(*n* = 11) −5.4 (9.7; −1.2)	(*n* = 9) −2.2 (−6.9;2.5)	−3.2 (−9.6;3.1)	(*n* = 14) −1.9 (−5.7;1.9)	(*n* = 16) −3.6 (−7.1; −0.1)	1.7 (−3.4;6.9)
FI ≥ 13.8 µIU/mL	(*n* = 19) −4.1 (−7.4; −0.9)	(*n* = 16) −3.7 (−7.3; −0.2)	−0.4 (−5.2;4.4)	(*n* = 15) −3.0 (−6.6;0.7)	(*n* = 17) −5.3 (−8.7; −1.8)	2.3 (−2.7;7.3)
HOMA-IR < 4.0	(*n* = 131) −3.4 (−4.6; −2.1) ^a^	(*n* = 130) −5.3 (−6.6; −4.1) ^b^	2.0 (0.2;3.7) *	(*n* = 115) −4.6 (−5.9; −3.3) ^ab^	(*n* = 133) −3.7 (−4.9; −2.5) ^ab^	−0.9 (−2.7;0.9)
HOMA-IR > 4.0	(*n* = 35) −2.8 (−5.2; −0.4) ^ab^	(*n* = 27) −4.5 (−7.2; −1.7) ^ab^	1.7 (−1.9;5.3)	(*n* = 33) −1.3 (−3.8;1.1) ^a^	(*n* = 35) −5.0 (−7.4; −2.5) ^b^	3.6 (0.2;7.1) *

Abbreviations: AP, Average protein; FI, Fasting insulin; FPG, Fasting plasma glucose; HOMA-IR, Homeostatic model assessment of insulin resistance; HF, High fat; HP, High protein; LF, Low fat. Data are presented as estimated mean weight changes from baseline for each combination of the diet × FPG × FI strata interaction in the linear mixed models, which were also adjusted for age, sex, and BMI (fixed effects) as well as sites (random effect). Differences in weight change from baseline between diets were compared within each blood marker group through the use of pairwise comparisons with post hoc *t* tests. Different superscript letters within a row indicate significant differences (*p* < 0.05). * *p* < 0.05; ^1^ Not adjusted for any fixed effects.

**Table 3:** Self-reported dietary fiber intake in the four randomized groups.

	LF-AP 65% Carb	LF-HP 55% Carb	HF-AP 45% Carb	HF-HP 34% Carb
Fiber g/10 MJ (month 6)	*n* = 80 36.6 ± 12.4 ^a^	*n* = 79 32.1 ± 10.8 ^b^	*n* = 68 32.1 ± 9.3 ^b^	*n* = 80 28.0 ± 9.9 ^c^
Fiber g/10 MJ (month 24)	*n* = 43 33.6 ± 12.8 ^a^	*n* = 46 29.1 ± 9.3 ^ab^	*n* = 41 28.6 ± 11.5 ^b^	*n* = 39 26.2 ± 10.2 ^b^
Fiber g/10 MJ (month 6 and 24) ^1^	*n* = 83 35.4 ± 11.7 ^a^	*n* = 81 31.4 ± 9.6 ^b^	*n* = 72 30.9 ± 9.2 ^b^	*n* = 81 27.3 ± 8.6 ^c^

Abbreviations: AP, Average protein; Carb, Carbohydrates; HF, High fat; HP, High protein; LF, Low fat. Data are mean ± SD and tested by one-way ANOVA with different superscript letters within a row indicate significant differences (*p* < 0.05). ^1^ Mean dietary fiber intake from month 6 and 24. If only one measurement was present this was used.

**Table 4:** Correlations between self-reported dietary fiber intake (g/10 MJ) and 24-month weight change (24-month − baseline) as well as weight change in groups of dietary fiber intake according to baseline glycemic and insulinemic status.

	*n*	Correlations ^1^ (Change in Fiber Intake ^2^)	Correlations ^1^ (Fiber Intake during Intervention ^3^)	*n*	Weight Change (kg) [<35 g Fiber/10 MJ ^3^]	*n*	Weight Change (kg) [≥35 g Fiber/10 MJ ^3^]	∆ Weight Change (kg) ^3^
All	317	−0.23 */−0.22 *	−0.17 */−0.18 *	210	−4.1 (−5.1;3.1)	107	−6.5 (−8.0; −4.9)	2.4 (0.6;4.1) *
FPG (mg/dL)								
<100	254	−0.21 */−0.21 *	−0.15 */−0.15 *	170	−4.0 (−5.1; −2.9)	84	−6.3 (−8.1; −4.5)	2.3 (0.3;4.3) *
≥100	63	−0.31 */−0.27 *	−0.25 */−0.27 *	40	−4.5 (−6.9; −2.0)	23	−7.1 (−10.2; −4.0)	2.7 (−1.3;6.6)
FI								
<13.8	210	−0.28 */−0.28 *	−0.23 */−0.22 *	138	−3.3 (−4.3; −2.3)	72	−6.0 (−7.7; −4.3)	2.7 (0.8;4.5) *
≥13.8	107	−0.15/−0.15	−0.12/−0.12	72	−5.6 (−7.8; −3.5)	35	−7.5 (−10.7; −4.3)	1.9 (−1.9;5.6)
FPG & FI								
<100 & <13.8	179	−0.24 */−0.24 *	−0.17 */−0.16 *	122	−3.4 (−4.5; −2.3)	57	−5.4 (−7.3; −3.6)	2.0 (0.02;4.0) *
<100 & ≥13.8	75	−0.15/−0.17	−0.14/−0.16	48	−5.6 (−8.2; −2.9)	27	−8.1 (−12.1; −4.1)	2.6 (−2.0;7.2)
≥100 & <13.8	31	−0.47 */−0.47 *	−0.45 */−0.47 *	16	−2.5 (−5.2;0.3)	15	−8.1 (−12.6; −3.5)	5.6 (0.6;10.6) *
≥100 & ≥13.8	32	−0.14/−0.20	−0.01/−0.05	24	−5.8 (−9.6; −2.0)	8	−5.4 (−9.2; −1.6)	−0.4 (−7.2;6.4)
HOMA-IR								
<4.0	251	−0.25 */−0.24 *	−0.19 */−0.19 *	167	−3.8 (−4.9; −2.8)	84	−6.5 (−8.2; −4.7)	2.6 (0.7;4.5) *
>4.0	66	−0.17/−0.16	−0.12/−0.10	43	−5.0 (−7.8; −2.3)	23	−6.5 (−9.7; −3.2)	1.4 (−3.0;5.8)

Abbreviation: FI, Fasting insulin; FPG, Fasting plasma glucose; HOMA-IR, Homeostatic model assessment of insulin resistance. ^1^ First number is Pearson correlation coefficients (r). Second number is partial correlation coefficients controlled for age, gender and baseline BMI. ^2^ Based on data from change in dietary fiber intake (g/10 MJ) (intervention – baseline). ^3^ Based on data from dietary fiber intake (g/10 MJ) during the intervention (not taking baseline dietary fiber intake into consideration). * *p* < 0.05.

## Supplementary Materials

The following are available online at https://www.mdpi.com/2072-6643/11/3/586/s1, Table S1: Two-year weight change according to randomization and stratified on pretreatment FPG and FI among subjects attending at least 44% (median value) of the counseling sessions (*n* = 319).
