# Supplementary material for: Pretreatment Fasting Glucose and Insulin as Determinants of Weight Loss on Diets Varying in Macronutrients and Dietary Fibers—The POUNDS LOST Study
Source: Nutrients. 2019 Mar 11;11(3):586. doi: 10.3390/nu11030586 (PMC6470525; doi:10.3390/nu11030586)
Supplement: Supplementary file 1 [file nutrients-11-00586-s001.pdf]

Table S1. Two year weight change according to randomization and stratified on pretreatment FPG and FI among subjects attending at least 44% (median value) of the counseling sessions (n=319).

	LF-AP 65% Carb	LF-HP 55% Carb	Δ (LF-AP vs. LF-HP) weight change (kg)	HF-AP 45% Carb	HF-HP 34% Carb	Δ (HF-AP vs. HF-HP) weight change (kg)
All[1]	(n=76) -5.9 (-7.5;-4.3)	(n=88) -7.2 (-8.7;-5.8)	1.3 (-0.7;3.4)	(n=71) -6.9 (-8.6;-5.3)	(n=84) -7.0 (-8.5;-5.5)	0.04 (-2.1;2.2)
FPG<100 mg/dL	(n=56) -5.4 (-7.2;-3.6)	(n=76) -7.5 (-9.1;-6.0)	2.1 (-0.2;4.4)	(n=58) -7.3 (-9.1;-5.5)	(n=65) -6.9 (-8.6;-5.3)	-0.3 (-2.7;2.1)
FI<13.8 μIU/mL	(n=36) -6.1 (-8.3;-3.9)	(n=57) -7.6 (-9.4;-5.9)	1.5 (-1.2;4.3)	(n=38) -6.9 (-9.0;-4.7)	(n=48) -6.6 (-8.5;-4.7)	-0.3 (-3.1;2.6)
FI≥13.8 μIU/mL	(n=20) -4.2 (-7.1;-1.2)	(n=19) -7.2 (-10.2;-4.1)	3.0 (-1.2;7.2)	(n=20) -8.1 (-11.1;-5.1)	(n=17) -8.0 (-11.2;-4.7)	-0.1 (-4.5;4.2)
FPG≥100 mg/dL	(n=20) -7.1 (-10.1;-4.1)	(n=12) -5.3 (-9.2;-1.5)	-1.8 (-6.6;3.0)	(n=13) -5.3 (-9.0;-1.6)	(n=19) -7.1 (-10.1;-4.0)	1.7 (-3.0;6.5)
FI<13.8 μIU/mL	(n=9) -7.0 (-11.4;-2.6)	(n=5) -3.6 (-9.4;2.3)	-3.4 (-10.7;3.9)	(n=7) -5.4 (-10.4;-0.5)	(n=10) -4.9 (-9.0;-0.7)	-0.5 (-7.0;5.9)
FI≥13.8 μIU/mL	(n=11) -7.2 (-11.2;-3.2)	(n=7) -6.6 (-11.5;-1.6)	-0.6 (-6.9;5.7)	(n=6) -5.2 (-10.5;0.2)	(n=9) -9.5 (-13.8;-5.1)	4.3 (-2.6;11.2)
HOMA-IR<4.0	(n=55) -6.2 (-8.0;-4.4)	(n=75) -7.2 (-8.7;-5.6)	1.0 (-1.3;3.3)	(n=58) -7.5 (-9.3;-5.7)	(n=66) -6.4 (-8.0;-4.7)	-1.1 (-3.5;1.2)
HOMA-IR>4.0	(n=21) -5.0 (-7.9;-2.1)[ab]	(n=13) -7.6 (-11.3;-4.0)[ab]	2.6 (-2.0;7.2)	(n=13) -4.4 (-8.0;-0.7)[a]	(n=18) -9.2 (-12.3;-6.0)[b]	**4.8 (0.01;9.6)***

Abbreviations: AP, Average protein; FI, Fasting insulin; FPG, Fasting plasma glucose; HOMA-IR, Homeostatic model assessment of insulin resistance; HF, High fat; HP, High protein; LF, Low fat. Data are presented as estimated mean weight changes from baseline for each combination of the diet x FPG x FI strata interaction in the linear mixed models, which were also adjusted for age, sex, and BMI (fixed effects) as well as sites (random effect). Differences in weight change from baseline between diets were compared within each blood marker group through the use of pairwise comparisons with post hoc t tests. Different superscript letters within a row indicate significant differences ($p < 0.05$). *P<0.05. [1]Not adjusted for any fixed effects.
